# An Update on Phosphodiesterase Mutations Underlying Genetic Etiology of Hearing Loss and Retinitis Pigmentosa

**DOI:** 10.3389/fgene.2018.00009

**Published:** 2018-02-08

**Authors:** Rahul Mittal, Nicole Bencie, James M. Parrish, George Liu, Jeenu Mittal, Denise Yan, Xue Zhong Liu

**Affiliations:** ^1^Department of Otolaryngology, University of Miami Miller School of Medicine, Miami, FL, United States; ^2^Department of Human Genetics, University of Miami Miller School of Medicine, Miami, FL, United States

**Keywords:** phosphodiesterase, CRISPR/Cas9, hearing loss, genetics, retinitis pigmentosa, usher syndrome

Cyclic nucleotide phosphodiesterases (PDEs) refer to a class of enzymes that degrade the phosphodiester bonds in cAMP and cGMP secondary messenger molecules (Tetsi et al., 2017; Weber et al., 2017). Due to their ability to regulate signal transduction, PDEs are vital in the modulation of biological signaling pathways in sensory systems including auditory and visual systems. The importance of PDEs in maintaining homeostatic environments in both the inner ear and the eye is highlighted by the fact that mutations in PDEs have been associated with various phenotypic abnormalities, including sensorineural hearing loss (SNHL), vision disorders, and other related syndromes (Bayés et al., 1995; Saga et al., 1998; Tetsi et al., 2017).

PDEs affect many biological systems (Fusco and Paldino, 2017), and PDE genetic mutations have been associated with the pathogenesis of several conditions, including sudden sensorineural hearing loss (SSNHL), retinitis pigmentosa (RP), and Usher Syndrome. While more research must be conducted and it is likely that the mechanisms surrounding SSNHL are complex, current data and several studies strongly supports the notion that PDE specifically PDE4D plays a role in causing SSNHL. PDE4D is expressed in the inner ear (Degerman et al., 2017) potentially playing a role in maintaining cochlear homeostasis and hearing function. In addition, PDE4Ds have been associated with atherosclerosis, which is a risk factor for SSNHL (Fischer et al., 2015; Rajati et al., 2016). While PDE4Ds are proposed as key players in SSNHL, PDE6 genes most likely play a role in the development of RP. PDE6 is an important component of visual phototransduction cascade (Majumder et al., 2015; Lagman et al., 2016) and therefore, low levels of PDE6 due to mutations can lead to rod-cone degeneration. This article will discuss the association of SSNHL, RP and Usher syndrome with PDE mutations and seek to elucidate the potential for common treatment modalities in such syndromes due to their shared etiologies.

## PDE mutations and hearing loss

The SNHL is defined as hearing loss resulting from damage to the inner ear or the auditory nerves that aid in the sound transducing mechanism to the brain (Luo et al., 2017). SSNHL is a form of SNHL that characterizes the sudden loss of hearing, usually in one ear and affects an estimated 5-27/100,000 persons per year (Alexander and Harris, 2013; Lin et al., 2017).

Until 2010, PDEs were known to be present in the cochlea and inner ear, but remained unidentified. Observation of the nitric oxide (NO) signaling pathway elucidated the existence and role that PDEs play in inner ear homeostasis. Most recently, PDE receptors were specified in the cochlea via reference to the guinea pig genome, which is up to 95% similar to the human genome. One study used the previously published full genome sequence of guinea pigs on the University of California, Santa Cruz (UCSC) genome bioinformatics browser to find PDEs present in humans, mice, and guinea pigs (Huang et al., 2010). The 22 PDE genes present in all three species were then located in guinea pig cochlear tissues using PCR. As a result, six PDE cDNA genes were identified in the guinea pig inner ear: *CpPde3a, CpPde4d, CpPde8a, CpPde8b, CpPde9a*, and *CpPde11a*. These genes were thus assumed to be present in the human ear based on the existence of cross-species gene superfamilies (Huang et al., 2010).

The identification of these PDEs in the cochlea led to studies that analyzed the correlations between genetic polymorphisms in these loci and SSNHL. In one Taiwanese study, mutations in the *PDE4D* gene were examined for their effects on the cochlea and possible pathogenic outcomes (Chien et al., 2016). As *PDE4D* mutations are known to cause ischemic stroke and cardiac abnormalities (Dichgans, 2007; Jørgensen et al., 2015; Shao et al., 2015) with associated hearing impairment, it was hypothesized that such mutations might play a role in the development of SSNHL. Results revealed a sex-differential effect of the *PDE4D* gene on SSNHL where only females had phenotypic disease symptoms (Chien et al., 2016). A rare T allele, coming from the rs702553 (SNP56) polymorphism of the *PDE4D* gene, increased prevalence of the condition in the female population. While studies have uncovered some correlations between the *PDE4D* gene and hearing function, the current lack of information warrants further study to better understand this relationship. A better knowledge about how PDE mutations affects cochlear function will help in developing novel treatment modalities for hearing loss.

## PDE mutations, retinitis pigmentosa and usher syndrome

Besides SNHL, retinitis pigmentosa (RP) demonstrates a heterogeneous genetic etiology. RP is a disorder characterized by gradual devolution of the retina and it affects between 1 in 3,000 and 8,000 people worldwide (Ali et al., 2017). The disorder can display X-linked, autosomal dominant or autosomal recessive inheritance patterns and has been associated with about 70 genes (Dias et al., 2017). The majority of such genes are expressed in photoreceptors of the pigment epithelium. The *PDE6* complex includes α, β, and γ subunits, which together, play an integral role in rod photoreceptor phototransduction (Majumder et al., 2015; Gopalakrishna et al., 2017). It has been demonstrated that *PDE6* maintains intracellular levels of cGMP by hydrolyzing cGMP when activated by light (Lagman et al., 2016). While the mechanism in RP that leads to rod photoreceptor death remains unknown, it is hypothesized to be a result of a defect in *PDE6* (Ali et al., 2017; Table 1).

**Table 1:** Phosphodiesterase (PDE) gene variants associated with retinitis pigmentosa.

**Gene**	**Function**	**Phenotype other than primary disease**	**Location**	**Mutation**	**dnSNP, ExAC, RCV (if available)**
*PDE6A*	Phototransduction	None	5q33.1	Tyr583Ter	dnSNP:rs121918576
					RCV000013989
				Ser344Arg	dbSNP:rs121918577
					RCV000013990
				Trp61Ter	dbSNP:rs121918578
					RCV000013991
				Val685Met	dbSNP:rs121909835
					ExAC:rs121909835
					RCV000022755
				IVS10AS, A-G,−2	dbSNP:rs1060499536
					RCV000210743
*PDE6B*	Phototransduction	Dominant congenital stationary night blindness	4p16.3	Gln298Ter	dbSNP:rs121918579
					ExAC:rs121918579
					RCV000013982
				Arg31Ter	dbSNP:rs121918580
					ExAC:rs121918580
					RCV000013983
				1-bp del, NT17981	dbSNP:rs730880317
					RCV000013984
				His557Tyr	dbSNP:rs121918581
					ExAC:rs121918581
					RCV000132576
					RCV000013985
				His258Asn	dbSNP:rs121918582
					RCV000013986
				71-BP DU	RCV000013987
				Trp807Arg	dbSNP:rs121918583; RCV000013988
				Arg60Cys	dbSNP:rs201541131; RCV000201856
*PDE6G*	Phototransduction	None	17q25.3	IVS3DS, G-T, +1	RCV000013981

For autosomal recessive RP (ARRP), over 40 genes have been mapped, including *PDE6A, PDE6B*, and *PDE6G* (RetNet, 2014). *PDE6A* and *PDE6B* are the second most common cause of ARRP, accounting for up to 2–5% of all such cases (Ali et al., 2017). While ARRP can be non-syndromic, it also presents as part of a constellation of signs in the form of a syndrome. Approximately 20–30% of ARRP patients have reported associations with non-ocular disorders. Usher syndrome is one such disease that includes both ARRP and hearing impairment. While Usher syndrome etiology is classically regarded as stemming from genes that do not contain *PDE6A, PDE6B*, or *PDE6G*, researchers have yet to identify whether the mechanisms that underlie vision loss in Usher syndrome are identical to those present in non-syndromic RP (Hmani-Aifa et al., 2009). Some patients diagnosed with Usher syndrome have been found to possess a so-called “double hit” of both classically regarded Usher mutations (e.g., *MYO7A, USH1C, CDH23*) in combination with *PDE6* mutations. This “double hit” not only appears to contribute to a greater severity of retinal devolution, but it also demonstrates the coexistence of SNHL, ARRP, and a mutation in *PDE*. The aforementioned genetic syndromes, along with a growing number of disorders that are associated with PDE, all make the case that a successful PDE-treatment modality might not only be applied to multiple disorders, but is certain to have vast interdisciplinary effects.

## Conclusion and future directions

While disorders such as SNHL, RP, and Usher syndrome encompass heterogeneous genetic etiologies, there is lack of information regarding *PDE*-related molecular mechanisms underlying each disease. There is a need to explore the tissue specificity of different PDEs and how PDE mutations lead to phenotypic consequences. In addition, the careful analysis of genetic etiology is imperative in diagnosis and development of new treatment modalities. The advent of new genome editing technologies including clustered regularly interspaced short palindromic repeats (CRISPR)/Cas9 holds a great potential as a therapeutic strategy for genetic diseases including SNHL and RP (Hsu et al., 2014; Zou et al., 2015). As a proof of this concept, CRISPR/Cas9 genome editing has been demonstrated to produce substantial improvement in mice with regard to retinal cone function and rod survival via the *NRL* gene (Peng et al., 2017; Yu et al., 2017). In the future, combining CRISPR/Cas9 with other powerful tools including adeno-associated virus (AAV) and iPSCs will open up avenues to develop effective therapeutic strategies to treat SNHL and RP having an underlying genetic etiology.

## Author contributions

All authors listed have made a substantial, direct and intellectual contribution to the work, and approved it for publication.

### Conflict of interest statement

The authors declare that the research was conducted in the absence of any commercial or financial relationships that could be construed as a potential conflict of interest.
